# Erste Klassifikationskriterien für durch Kalziumpyrophosphatablagerungen verursachte Erkrankungen – Übersetzung, Erläuterung und Bewertung

**DOI:** 10.1007/s00393-024-01482-2

**Published:** 2024-02-21

**Authors:** Jürgen Braun, Martin Krekeler, Uta Kiltz

**Affiliations:** 1 Rheumatologisches Versorgungszentrum Steglitz, Berlin, Deutschland; 2 Ruhr Universität Bochum, Bochum, Deutschland; 3 Rheumazentrum Ruhrgebiet, Claudiusstr. 45, 44649 Herne, Deutschland

**Keywords:** ACR/EULAR Klassifikationskriterien 2023, Crowned-Dens-Syndrom, Analyse der Synovialflüssigkeit, Kristalle, Chondrokalzinose, ACR/EULAR classification criteria 2023, Crowned dens syndrome, Synovial fluid analysis, Crystals, Chondrocalcinosis

## Abstract

**Zielsetzung:**

Für die durch Kalziumpyrophosphatablagerungen verursachten Erkrankungen („calcium pyrophosphate deposition [CPPD] disease“) fehlten bislang validierte Klassifikationskriterien. Die kürzlich hierfür entwickelten und validierten Klassifikationskriterien wurden in dieser Arbeit übersetzt, erläutert und bewertet.

**Methoden:**

In den letzten Jahren hat eine multinationale Forschergruppe mit Unterstützung der European Alliance of Associations for Rheumatology (EULAR) und dem American College of Rheumatology (ACR) Klassifikationskriterien für die CPPD-Erkrankung einer etablierten Methodik folgend entwickelt. Die Übersetzung und Kommentierung der neuen ersten Klassifikationskriterien für die CPPD-Erkrankung erfolgten iterativ im Konsens der Autoren.

**Ergebnisse:**

Für die Klassifikation als CPPD-Erkrankung reicht das Vorhandensein eines Crowned-Dens-Syndroms oder von Kalziumpyrophosphatkristallen in der Synovialflüssigkeit bei Patienten mit Gelenkschmerzen, -schwellungen oder -empfindlichkeit (Eintrittskriterium), deren Symptome nicht vollständig durch eine andere rheumatische Erkrankung erklärt werden können (Ausschlusskriterium), aus, um eine(n) Betroffene(n) als CPPD-Patient*in zu klassifizieren. Liegen diese Befunde nicht vor, kann eine Punktzahl von mehr als 56 Punkten anhand gewichteter Kriterien, die sich aus klinischen Merkmalen, Ergebnissen und Befunden zusammensetzen, zur Einstufung als CPPD-Erkrankung herangezogen werden. Diese Kriterien hatten eine Sensitivität von 92,2 % und eine Spezifität von 87,9 % in der Ableitungskohorte (190 CPPD-Fälle, 148 Mimiker), während die Sensitivität 99,2 % und die Spezifität 92,5 % in der Validierungskohorte (251 CPPD-Fälle, 162 Mimiker) betrug.

**Schlussfolgerung:**

Die ACR/EULAR-Klassifikationskriterien von 2023 für die CPPD-Erkrankung werden die klinische Forschung auf diesem Gebiet erleichtern. Die klinische Anwendung i wird zeigen, wie praktikabel die Kriterien sind.

## Abstract (en)

**Aim:**

For diseases caused by calcium pyrophosphate deposition (CPPD), validated classification criteria were previously lacking. In this article the recently developed and validated classification criteria are translated, explained, and assessed.

**Methods:**

In recent years a multinational research group developed classification criteria for CPPD disease with the support by the European Alliance of Associations for Rheumatology (EULAR) and the American College of Rheumatology (ACR), following an established method. The developed criteria were finally validated in an independent cohort. The translation and annotation of the new first classification criteria were carried out in an iterative procedure in consensus with the authors.

**Results:**

The presence of a crowned dens syndrome or calcium pyrophosphate crystals in the synovial fluid in patients with pain, swelling or sensitivity of the joints (entry criterion) is sufficient for the classification as CPPD disease, where the symptoms cannot be completely explained by another rheumatic disease (exclusion criterion). If these symptoms are not present, a count of more than 56 points based on weighted criteria comprised of clinical features and the results of laboratory and imaging investigations can be included for classification as a CPPD disease. These criteria had a sensitivity of 92.2% and a specificity of 87.9% in the derivation cohorts (190 CPPD cases and 148 mimics), whereas the sensitivity was 99.2% and the specificity 92.5% in the validation cohorts (251 CPPD cases and 162 mimics).

**Conclusion:**

The ACR/EULAR classification criteria 2023 of a CPPD disease will facilitate clinical research in this field. The use in the clinical routine will show how practical the criteria are.

Die durch die Ablagerung von Kalziumpyrophosphatkristallen („calcium pyrophosphate deposition“ [CPPD]) bedingte symptomatische Arthritis, die auch als Pseudogicht bezeichnet wird, ist durch verschiedene klinische Merkmale gekennzeichnet [1, 2]. Die Prävalenz der röntgenologischen Chondrokalzinose, die häufig (nicht korrekterweise) als Ersatz für die CPPD-Erkrankung verwendet wird, liegt bei älteren Erwachsenen zwischen 4 und ≥10 %, während die Prävalenz der symptomatischen CPPD-Erkrankung nach wie vor nicht sicher anzugeben ist [3–6]. Die Forschung im Bereich der CPPD-Erkrankung ist im Vergleich zu anderen Arthritiden in den letzten Jahrzehnten eher begrenzt geblieben – was zum Teil auf das Fehlen geeigneter Klassifikationskriterien (Abb. 1) zurückzuführen ist. So wird der wissenschaftliche Vergleich von Studiendaten unter anderem durch die die Vielfalt der Erscheinungsformen der CPPD-Erkrankung, zu denen die akute und chronisch entzündliche CPPD-Kristallarthritis, die Osteoarthritis mit CPPD und das Crowned-Dens-Syndrom (CDS) gehören, und die lange historische Abhängigkeit der Diagnosestellung von der Polarisationslichtmikroskopie der Synovialflüssigkeit (SF) erschwert [2]. Die einzigen bisher veröffentlichten Diagnosekriterien für die CPPD-Erkrankung wurden in den 1960er-Jahren entwickelt [7]. Für eine eindeutige Diagnose verlangten sie darin den Nachweis von Kristallen auf der Grundlage des Vorhandenseins von sowohl typischen Verkalkungen auf dem Röntgenbild als auch von Befunden, die mit dem Nachweis von CPPD in der SF-Polarisationslichtmikroskopie (Abb. 2) übereinstimmen [8]. Diese Diagnosekriterien wurden inzwischen als problematisch erkannt, da die konventionelle Radiographie eine geringe Sensitivität für CPPD [9–11] und die SF-Analyse für CPPD eine zu hohe Falsch-negativ-Rate und darüber hinaus eine ziemlich hohe Variabilität zwischen den Bewertern aufweist [12–15]. Des Weiteren standen in den 1960er-Jahren moderne Bildgebungsverfahren wie Ultraschall (Abb. 3 und Abb. 4) und Dual-Energie-Computertomographie (DECT), die eine höhere Sensitivität für CPPD-assoziierte Veränderungen aufweisen, noch nicht zur Verfügung ([12–15], s. Abb. 4).

**Figure 1:**
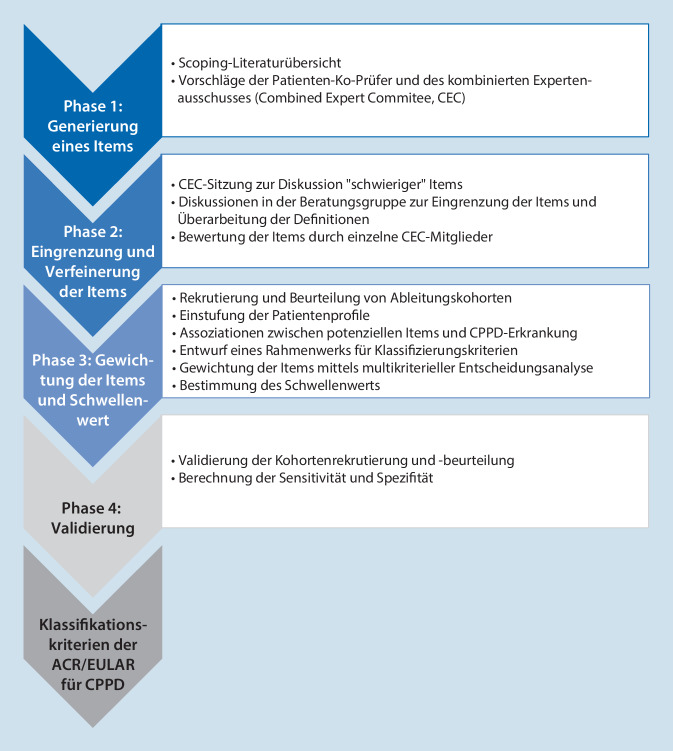

**Figure 2:**
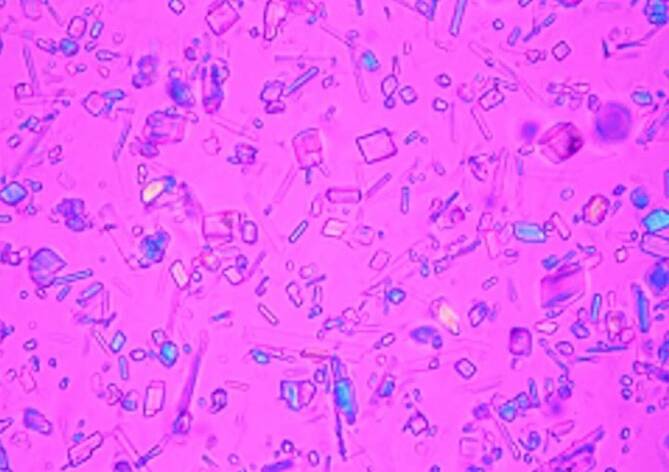

**Figure 3:**
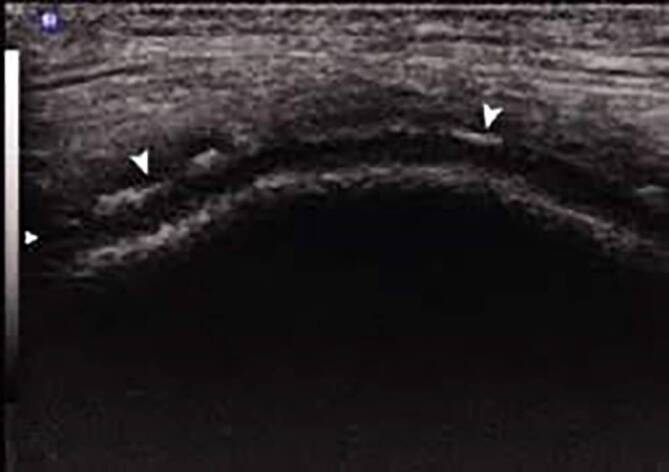

**Figure 4:**
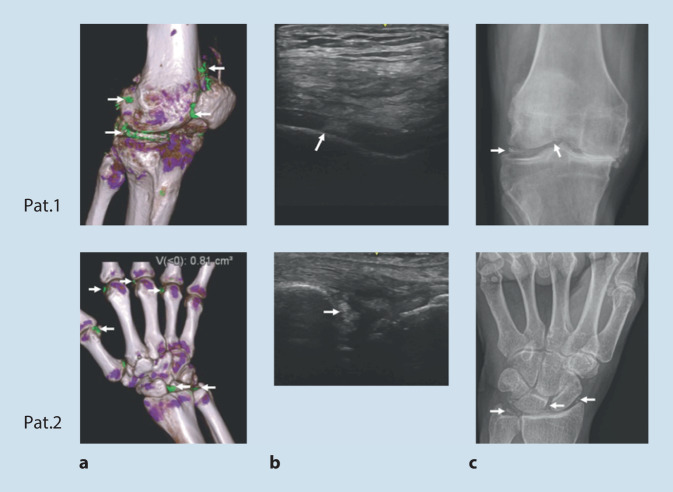

Um validierte Klassifikationskriterien (Tab. 1) zu entwickeln, die die Erforschung der CPPD-Krankheit erleichtern sollen, wurde mit Unterstützung des American College of Rheumatology (ACR) und der European Alliance of Associations for Rheumatology (EULAR) eine internationale Arbeitsgruppe einberufen. Das Ziel war die Entwicklung von Klassifikationskriterien, die es den Forschern ermöglicht, Menschen mit CPPD-Erkrankungen für die Teilnahme an klinischen und Beobachtungsstudien sicherer zu identifizieren [16, 17].

**Table 1:** 

**Definition der Kriterien**		
*Die Klassifikationskriterien für die CPPD-Erkrankung sollten in folgender Reihenfolge angewendet werden*		
1	Einstiegskriterium: Mindestens eine Episode von Gelenkschmerzen, -schwellungen oder Druckempfindlichkeiten in der Vergangenheit an einem peripheren Gelenk oder im Achsenskelett	
2	Absolute Ausschlusskriterien: Alle Symptome, die sich mit größerer Wahrscheinlichkeit durch eine andere Erkrankung erklären lassen (z. B. rheumatoide Arthritis, Psoriasisarthritis, Lupus, Gicht, PMR, OA usw.). Siehe auch entsprechende Klassifikationskriterien [18–22]	
3	Ausreichende Kriterien: Nachweis eines Crowned-Dens-Syndroms oder von CPP-Kristallen in der Synovialflüssigkeit in einem Gelenk mit Schwellung, Berührungsempfindlichkeit oder Schmerzen^a^	
**Domänen**		**Punkte**
A	*Alter bei Auftreten von Gelenksymptomen (Schmerzen, Schwellungen und/oder Druckempfindlichkeit)*	
	≤60 Jahre	0
	>60 Jahre	4
B	*Zeitlicher Verlauf und Symptome der Arthritis*^*b*^	
	Keine persistierende oder typische Arthritis	0
	Persistierende Arthritis	9
	Ein typischer akuter Arthritisschub	12
	Mehr als eine typische akute Arthritisepisode	16
C	*Lokalisation(en) typischer Episoden Arthritis in peripheren Gelenken*	
	MTP-1-Gelenk	−6
	Keine typische(n) Episode(n)	0
	Andere(s) Gelenk(e) als Handgelenk, Knie oder MTP-1-Gelenk	5
	Handgelenk	8
	Kniegelenk	9
D	*Verwandte Stoffwechselkrankheiten*^c^	
	Keine	0
	Vorhanden	6
E	*Analyse von Kristallen in der Synovialflüssigkeit aus einem symptomatischen Gelenk*	
	CPP-Kristalle bei ≥2 Untersuchungen nicht vorhanden	−7
	CPP-Kristalle in 1 Untersuchung nicht vorhanden	−1
	Untersuchung nicht durchgeführt	0
F	*Osteoarthrose der Hand/des Handgelenks in der Bildgebung (definiert als vorhanden, wenn der K/L-Score ≥2 ist)*	
	Keiner der unten aufgeführten Befunde oder keine Bildgebung des Handgelenks/der Hand durchgeführt	0
	OA der Radiokarpalgelenke beidseitig	2
	≥2 der folgenden Befunde: STT-Gelenk-OA ohne OA des ersten CMC-Gelenks; OA des 2. MCP-Gelenks; OA des 3. MCP-Gelenks	7
G	*Bildgebender Nachweis von CPPD in symptomatischen peripheren Gelenken*^d^	
	Keine Nachweis in US, CT oder DECT (und fehlend auf CR/nicht durchgeführt)	−4
	Keine bei CR (und US, CT, DECT nicht durchgeführt)	0
	Vorhanden entweder bei CR, US, CT oder DECT	6
H	*Anzahl der peripheren Gelenke mit Anzeichen von CPPD mit einem beliebigen bildgebenden Verfahren, unabhängig von den Symptomen*^d^	
	Keine	0
	1	16
	2–3	23
	≥4	25

Die zahlreichen Fußnoten sind extensiv erklärt und hier aufgeführt:

Zu den klinischen Merkmalen gehören akut oder subakut auftretende starke, auf den oberen Halsbereich beschränkte Schmerzen mit erhöhten Entzündungsmarkern, eingeschränkter Rotation und häufig Fieber. Erkrankungen mit einer ähnlichen Symptomatik wie Polymyalgia rheumatica und Meningitis sollten ausgeschlossen worden sein

In der konventionellen Computertomographie (CT) lassen sich Kalkablagerungen im transversalen Atlasband erkennen, die typischerweise linienförmig sind und eine geringere Dichte als die des kortikalen Knochens aufweisen. In der axialen Ansicht sehen diese oft wie 2 parallele Linien aus. Charakteristisch sind auch Verkalkungen im atlantoaxialen Gelenk, im Ligamentum alare und/oder eine Pannusformation an der Spitze des Dens

Zu den Merkmalen der Dual-Energy-Computertomographie (DECT) gehört ein Dual-Energy-Index zwischen 0,016 und 0,036. Sowohl die klinischen als auch die bildgebenden Merkmale müssen vorhanden sein. Kriterien für einen positiven Befund sind auch erfüllt, wenn bei der histopathologischen Analyse des Gelenkgewebes CPP-Kristalle nachgewiesen werden – vorausgesetzt, dass nicht bereits die Ausschlusskriterien erfüllt sind. So kann z. B. die Ablagerung von CPP-Kristallen im Gelenkknorpel von Patienten mit OA im Endstadium nicht dazu verwendet werden, den Patienten als Patienten mit CPPD zu klassifizieren, wenn alle Symptome besser durch das Vorhandensein von OA erklärbar sind (Ausschlusskriterien)

Eine persistierende Arthritis wurde als anhaltende Gelenkschwellung mit Schmerzen und/oder Erwärmung von ≥1 Gelenk(en) definiert. Eine typische Krankheitsepisode wurde definiert als eine Episode mit akutem Beginn oder akuter Verschlimmerung von Gelenkschmerzen mit Schwellung und/oder Erwärmung, die unabhängig von Behandlung wieder verschwindet

*CMC* karpometakarpal, *CR* konventionelle Radiographie, *CT* Computertomographie, *K/L* Kellgren/Lawrence, *MCP* metakarpophalangeal, *MTP* metatarsophalangeal, *STT* skaphotrapeziotrapezoid, *US* Ultraschall

^a^Episode, die in einem peripheren Gelenk oder im Falle des Crowned-Dens-Syndroms in einem axialen Gelenk wie C1/C2 auftritt

^b^Das Crowned-Dens-Syndrom ist definiert durch das Vorhandensein von (a) klinischen Merkmalen und (b) bildgebenden Befunden

^c^Einschließlich der hereditären Hämochromatose, des primären Hyperparathyreoidismus, der Hypomagnesiämie, des Gitelman-Syndroms, der Hypophosphatasie oder einer familiären Vorgeschichte einer CPPD-Erkrankung. Analysen der Synovialflüssigkeit sollten von Personen durchgeführt werden, die in der Anwendung von Polarisationsmikroskopie geschult sind

^d^Eine Bildgebung mindestens eines symptomatischen peripheren Gelenks mittels CR, US, CT oder DECT ist erforderlich, um für die Klassifikation in Betracht gezogen zu werden, wenn keine ausreichenden Kriterien erfüllt sind. Der bildgebende Nachweis einer CPPD bezieht sich auf eine Verkalkung des Faserknorpels oder des hyalinen Knorpels. Verkalkungen der Synovialmembran, der Gelenkkapsel oder der Sehne dürfen nicht bewertet werden. Bildgebende Definitionen sind an anderer Stelle veröffentlicht [23–27]. Nur die Beteiligung peripherer Gelenke wird berücksichtigt

## Methoden

**Figure 5:**
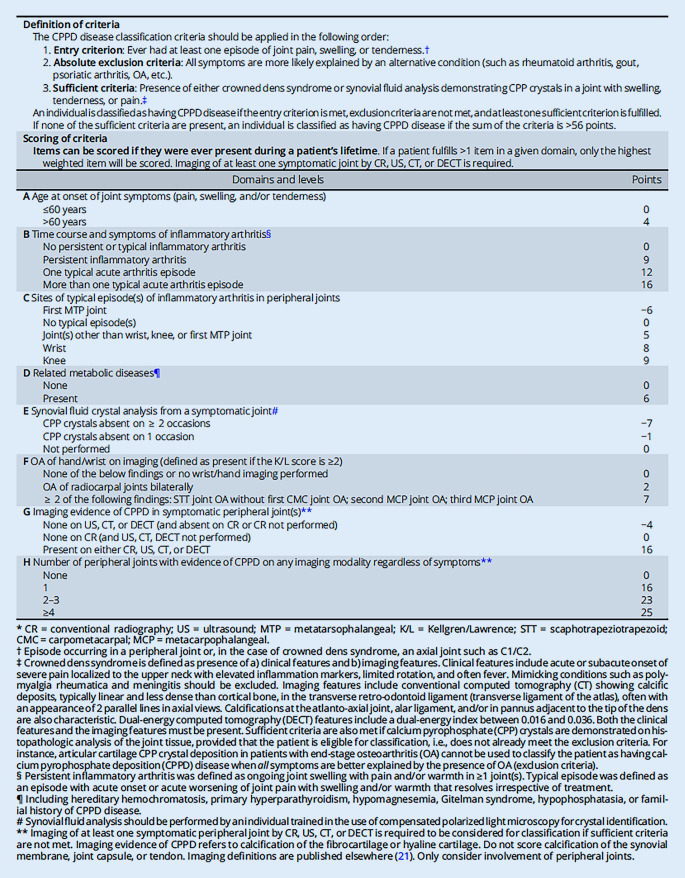

Die detaillierte Beschreibung der Entwicklung der Klassifikationskriterien für die CPPD-Erkrankung ist der Originalpublikation zu entnehmen (Abb. 5; [16, 17]). Diese gliederte sich in die Erstellung von Variablenlisten, die Identifikation von Patientenprofilen und die Untersuchung der Assoziationen zwischen den Variablen und der CPPD-Erkrankung. In einer Entscheidungsanalyse wurden unter Berücksichtigung aller Faktoren Kriterien gewichtet und Schwellenwerte definiert. Die entwickelten Kriterien wurden dann in einer unabhängigen Kohorte validiert.

Die Übersetzung und Kommentierung der neuen ersten Klassifikationskriterien für die CPPD-Erkrankung erfolgte in einem iterativen Verfahren gemeinsam im Konsens der Autoren.

## Ergebnisse

**Figure 6:**
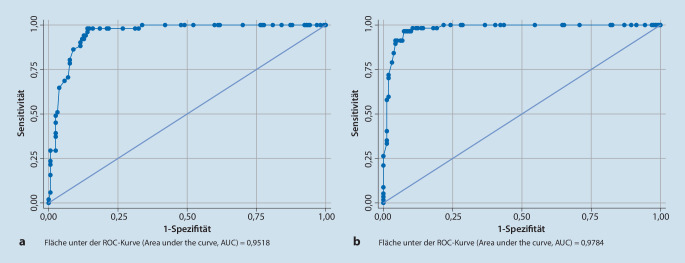

Die multinationale Forschergruppe hat für die Klassifikation als CPPD-Erkrankung verschiedene Kriterien definiert. Für die Klassifikation als CPPD-Erkrankung reicht das Vorhandensein von Kalziumpyrophosphatkristallen in der Synovialflüssigkeit oder der Nachweis eines Crowned-Dens-Syndroms bei Patienten mit Gelenkschmerzen, -schwellungen und/oder Druckempfindlichkeit (Eintrittskriterium), deren Symptome nicht vollständig durch eine andere rheumatische Erkrankung erklärt werden können (Ausschlusskriterium), aus, um eine(n) Betroffene(n) als CPPD-Patient*in zu klassifizieren. Liegen diese Befunde nicht vor, kann eine Punktzahl von mehr als 56 Punkten anhand gewichteter Kriterien, die sich aus klinischen Merkmalen, begleitenden Stoffwechselstörungen und Ergebnissen von Labor- und Bildgebungsuntersuchungen zusammensetzen, zur Klassifikation als CPPD-Erkrankung herangezogen werden. Diese Kriterien hatten eine Sensitivität von 92,2 % und eine Spezifität von 87,9 % in der Entwicklungskohorte (190 CPPD-Fälle, 148 CPPD „mimics“), während die Sensitivität 99,2 % und die Spezifität 92,5 % in der Validierungskohorte (251 CPPD-Fälle, 162 CPPD „mimics“) betrug ([17]; s. auch Abb. 6).

## Diskussion

Diese ersten Klassifikationskriterien für die CPPD-Erkrankung [17] haben insgesamt ein gutes Potenzial, die klinische Forschung auf diesem Gebiet zu beflügeln. Die validierten Kriterien wurden mithilfe einer etablierten Methodik auf der Basis eines fundierten Expertenkonsens entwickelt, dabei standen Daten von 751 Patientenprofilen zur Verfügung. In einer unabhängigen Validierungskohorte zeigten sich eine hohe Sensitivität und Spezifität.

Das Vorhandensein von bildgebenden Verfahren in Kombination mit klinischen Merkmalen oder die Identifizierung von CPP-Kristallen in der SF aus einem symptomatischen Gelenk reichen für die Klassifikation einer CPPD-Erkrankung aus, wenn keine Ausschlusskriterien erfüllt sind (z. B. wenn eine andere Erkrankung die gesamte klinische Präsentation schlüssig erklären kann).

Unter den bewerteten Kriterien haben bildgebende Merkmale und wiederkehrende typische Episoden einer akuten entzündlichen Arthritis das größte Gewicht. Die multidisziplinären Experten waren sich letztlich einig, dass der bildgebende Nachweis von CPP-Kristallablagerungen und die akute entzündliche Arthritis zentrale Konstrukte der CPPD-Erkrankung sind, wenn der Labornachweis von typischen CPP-Kristallen in der Synovialflüssigkeit fehlt.

Bei Patienten, die diese klinischen Kriterien nicht erfüllen, ist eine bildgebende Untersuchung von mindestens einem symptomatischen Gelenk erforderlich. Je mehr periphere Gelenke abgebildet werden, desto höher ist die potenziell erreichbare Punktzahl – dies ist z. B. in Zentren der Fall, in denen die Gelenke routinemäßig beidseitig untersucht werden. Grundsätzlich eine standardisierte Bildgebung von bestimmten Gelenken (z. B. beide Knie und Handgelenke) zu verlangen, wurde nur diskutiert, aber nicht mit aufgenommen. Die Bildgebung von mindestens einem symptomatischen peripheren Gelenk stellt einen vernünftigen Kompromiss im Rahmen der Klassifikation dar. Das kann im Rahmen der Diagnosestellung aber durchaus anders aussehen.

Die Klassifikationskriterien betonen die Bedeutung des bildgebenden Nachweises von CPPD, auch wenn bildgebende Merkmale allein bei einem Patienten mit Arthralgien für eine Klassifikation CPPD-Erkrankung nicht ausreichen würden. Die höchsten Werte in 2 bildgebenden Bereichen machen fast die Hälfte der Gewichtung aus: der Nachweis von CPP-Kristallen in einem symptomatischen Gelenk und in ≥ 4 peripheren Gelenken. Die hohe Sensitivität von Ultraschall und CT, insbesondere im Frühstadium einer CPPD-Erkrankung, im Vergleich zu CR führte zur Vergabe von Negativpunkten, wenn in der fortgeschrittenen Bildgebung kein Hinweis auf eine CPPD-Erkrankung gefunden wird [11, 23]. Da die fortgeschrittenen Techniken eine hohe, aber nicht vollkommene Spezifität für die CPPD-Erkrankung aufweisen, konnte man sich aber nicht darauf einigen, dass der Nachweis einer CPPD in der fortgeschrittenen Bildgebung ausreicht, um eine Klassifikation als CPPD-Erkrankung zu erreichen. Der bildgebende Nachweis von CPPD mit fortgeschrittenen bildgebenden Verfahren oder CR wurde angesichts der hohen Spezifität beider Verfahren nahezu gleich gewichtet.

Ein praktischer diagnostischer Goldstandard für die CPPD-Erkrankung existiert in der klinischen Praxis nicht, da die Positivität von CPP-Kristallen in der Polarisationsmikroskopie der SF zwar spezifisch ist, aber eine hohe Falsch-negativ-Rate und eine erhebliche Variabilität zwischen den Beobachtern aufweist [12–15]. Zu den Herausforderungen bei der Identifizierung von CPP-Kristallen gehören die geringe Größe der Kristalle und die fehlende bzw. nur schwach positive Doppelbrechung [12]. Darüber hinaus kann die Durchführbarkeit der Identifizierung von CPP-Kristallen durch die Schwierigkeit der Gelenkaspiration, insbesondere aus kleinen Gelenken, eingeschränkt sein. Obwohl das Vorhandensein einer beliebigen Menge von CPP-Kristallen in einem symptomatischen Gelenk zur Einstufung als CPPD-Erkrankung führen kann, ist die Forderung nach dem Vorhandensein von CPP-Kristallen in der Synovialflüssigkeit in allen Fällen für die Klassifikation nicht praktikabel. Daher sollen die vorgeschlagenen Kriterien eine Klassifikation als CPPD-Erkrankung unabhängig davon ermöglichen, ob eine Gelenkpunktion durchgeführt wurde. Dennoch bleibt diese wie bei der Gicht wichtig, um eine CPPD-Erkrankung in der Praxis sicher zu diagnostizieren und um andere Erkrankungen wie septische Arthritis auszuschließen.

Die Zuordnung von Symptomen zu einer CPPD-Erkrankung kann schwierig sein, insbesondere bei Patienten mit Osteoarthrose (OA) oder rheumatoider Arthritis (RA), da diese Krankheiten mit der CPPD-Erkrankung koexistieren [28] und/oder fehldiagnostiziert werden können [1, 29, 30]. Die hier vorgestellten Klassifikationskriterien für die CPPD-Erkrankung tragen der häufigen Koexistenz dieser Erkrankung mit anderen rheumatischen und muskuloskeletalen Erkrankungen Rechnung, indem sie nur solche Patienten von der Klassifikation ausschließen, deren Symptome besser durch eine andere Erkrankung erklärt werden können. Die Unterscheidung zwischen CPP-Kristallablagerungen und einfachen Kalzifikationen in bildgebenden Verfahren kann schwierig sein, obwohl die im Rahmen dieses Projekts entwickelten bildgebenden Definitionen für die CPPD-Erkrankung dieses Problem minimieren könnten [23].

**Figure 7:**
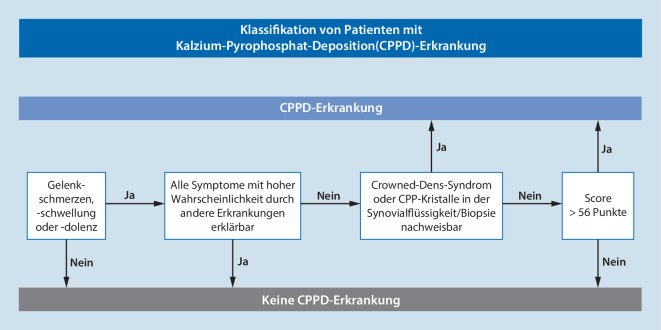

Die von den Autoren benannten Stärken dieser ersten Klassifikationskriterien für die CPPD-Erkrankung bestehen darin, dass das klinische Bild als entzündliche Arthritis bei älteren Erwachsenen festgelegt wurde, die sich typisch und vorzugsweise an Knie- und Handgelenken entzündlich manifestiert (Prädilektionsstellen), wobei meist auch erhöhte Entzündungsparameter vorliegen. Das Erfordernis des Vorliegens einer Gelenkentzündung geht mit hoher Spezifität und Sensitivität von mehr als 90 % bei Patienten ohne Nachweis in der Bildgebung oder ohne Nachweis von Kristallen in der SF einher. Bei Personen mit OA und CPP-Kristallen in der SF ist eine entzündliche Arthritis nicht unbedingt erforderlich, denn diese könnten mit den vorliegenden Kriterien klassifiziert werden, wenn nicht alle Symptome durch die OA erklärt werden können. Entscheidend ist, dass die Klassifikationskriterien in der in Abb. 7 und Tab. 1 dargestellten Reihenfolge angewendet werden, sodass Personen, deren Symptome auf OA zurückzuführen sind und die CPP-Kristalle in der SF aufweisen, nicht als CPPD-Patienten klassifiziert werden können. Darüber hinaus ist zu betonen, dass die Patientenprofile in den Ableitungs- und Validierungskohorten aus einem großen internationalen Pool stammten, was die Verallgemeinerbarkeit der Ergebnisse unterstützt. Dennoch ist eine weitere Prüfung der Kriterien in anderen Populationen grundsätzlich sinnvoll. Wir prüfen gerade, ob dies in unserem kürzlich publizierten Datensatz möglich ist [30]. Weiterhin besteht eine Stärke der Kriterien darin, dass die multidisziplinäre Gruppe einer etablierten Methodik für die Entwicklung von Klassifikationskriterien gefolgt ist, was die Validität des Prozesses und des Endprodukts unterstützt. Gesichert ist, dass die Kriterien eine Klassifikation von Patient*innen mit und ohne CPPD-Erkrankung erlauben.

In der Diskussion wurden auch einige Einschränkungen erwähnt: In Abwesenheit eines diagnostischen Goldstandards für die Diagnose einer CPPD-Erkrankung wurden Fälle und Mimikry-Patienten nach Expertenmeinung eingestuft. Initial wurde eine beträchtliche Anzahl unsicherer Patientenprofile aus den ROC-Analysen und Sensitivitäts‑/Spezifitätsberechnungen ausgeschlossen, da ihr tatsächlicher Fall‑/Kontrollstatus nicht zuverlässig bestimmt und zugeordnet werden konnte. Die Heterogenität der CPPD-Erkrankung kann bekanntermaßen zu unterschiedlichen klinischen Einschätzungen in Bezug auf die richtige Diagnose führen. Dies spiegelt sich auch in der Bewertung der Wahrscheinlichkeit einer CPPD-Erkrankung durch erfahrene Kliniker bzw. in der mangelnden Übereinstimmung zwischen Beurteilern wider. Durch das heterogene klinische Erscheinungsbild der Erkrankung variiert die Wahrnehmung des klinischen Phänotyps, der einer CPPD-Erkrankung zugeschrieben werden kann, bei Rheumatologen erheblich. Um die Möglichkeit zu minimieren, dass sich dies auf die Festlegung der Schwellenwerte auswirkt, wurden strenge Fall- und Mimiker-Definitionen festgelegt, die den eindeutigen Nachweis einer CPPD-Erkrankung und eine solide Übereinstimmung zwischen einreichendem Arzt und 2 Experten erforderten. Dass nur eindeutige Fälle und eindeutige Mimiker in die Analyse einbezogen wurden, hat wahrscheinlich zu der hohen Sensitivität und Spezifität der Klassifikationskriterien in der Validierungskohorte beigetragen. Nichtsdestotrotz stieg der Anteil der Personen, die als an CPPD erkrankt eingestuft wurden, mit der Einstufung des einreichenden Klinikers zunehmend an, was die interne Validität dieses Ansatzes unterstützt. Trotz der Schwierigkeiten bei der Zuordnung ermöglichen die CPPD-Klassifikationskriterien die Identifizierung einer relativ homogenen Gruppe von Patienten mit überwiegendem Nachweis von CPP-Kristallablagerungen und charakteristischen klinischen Symptomen, bei denen die vorliegenden Merkmale nicht besser durch eine andere Erkrankung erklärt werden können.

Wichtig zu betonen ist noch, dass die asymptomatische CPPD, also z. B. der radiologische Befund einer asymptomatischen Chondrokalzinose, von vornherein bewusst nicht Thema der Expertengruppe war, da der wesentliche Zweck der Klassifikationskriterien ja darin besteht, Personen mit einer symptomatischen Erkrankung zu identifizieren, die dann auf dieser Basis in klinische Studien aufgenommen werden können. Die aktuellen Kriterien hatten ja das klare Ziel, Patienten mit symptomatischer CPPD-Erkrankung mit maximaler Sensitivität und Spezifität korrekt zu klassifizieren.

Aus unserer Sicht sind die Kriterien insgesamt gelungen, wobei die Komplexität der Kriterien – es sind ja 8 Bereiche zu berücksichtigen, wobei 0 bis 83 Punkte zu erreichen sind – der Komplexität des klinischen Problems geschuldet ist. Abzuwarten bleibt, wie praktikabel der Einsatz der Kriterien im klinischen Alltag sein wird.

Zusammenfassend lässt sich sagen, dass die aktuell 2023 vorgestellten ACR/EULAR-Klassifikationskriterien für die CPPD-Erkrankung den ersten validierten Kriteriensatz für diese Erkrankung mit solide validierten Leistungsmerkmalen darstellen. Diese Kriterien können zukünftig als Einschlusskriterien für die Rekrutierung von Studienteilnehmer*innen verwendet werden.
